# Claudin-4 is required for vasculogenic mimicry formation in human breast cancer cells

**DOI:** 10.18632/oncotarget.3571

**Published:** 2015-03-14

**Authors:** Yong-Feng Cui, An-Heng Liu, Dai-Zhi An, Ru-Bao Sun, Yun Shi, Yun-Xiang Shi, Miao Shi, Qiang Zhang, Li-Li Wang, Qiong Feng, Gui-Lan Pan, Qiang Wang

**Affiliations:** ^1^ Center of Hygiene Assessment and Research, Institute of Disease Control and Prevention, Academy of Military Medical Sciences, Beijing, China; ^2^ Cardiovascular Medicine, Affiliated Hospital of Academy of Military Medical Sciences, Beijing, China; ^3^ Department of Physiology, BaoTou Medical College, Inner Mongolia University of Science and Technology, Baotou, China

**Keywords:** claudins, tight junction, vasculogenic mimicry, aggressive breast cancer

## Abstract

Vasculogenic mimicry (VM) refers to the unique capability of aggressive tumor cells to mimic the pattern of embryonic vasculogenic networks. Claudins are aberrantly expressed in aggressive breast cancer. However, the relationship between claudins and VM formation is not clear. We examined VM in two human breast cancer cell lines with different aggressive capabilities (MDA-MB-231 and MCF-7 cells) and one human umbilical vein endothelial cell line (HUVEC). Both HUVEC and MDA-MB-231 cells formed vascular channels in Matrigel cultures, while MCF-7 cells did not. Western blot analysis revealed a possible correlation between claudin-4 and -6 expression in breast cancer cell lines and tumor aggressiveness, with protein levels correlating with the ability to form vascular channels. Treatment of MDA-MB-231 and HUVEC cells with claudin-4 monoclonal antibodies completely inhibited the ability of cells to form vascular channels. Moreover, knockdown of claudin-4 by short hairpin RNA completely inhibited tubule formation in MDA-MB-231 cells. Overexpression of claudin-4 in MCF-7 cells induced formation of vascular channels. Immunocytochemistry revealed that membranous claudin-4 protein was significantly associated with vascular channel formation. Collectively, these results indicate that claudin-4 may play a critical role in VM in human breast cancer cells, opening new opportunities to improve aggressive breast cancer therapy.

## INTRODUCTION

Breast cancer is the most common malignancy in women and is the leading cause of death among women world-wide [1]. Despite many advances in the diagnosis and treatment of breast cancer, metastasis remains an insurmountable challenge. Although the molecular mechanisms are not completely understood, it is well established that the formation and growth of new blood vessels is critical for sustained tumor growth and metastasis [2]. Studies have found that benign lesions associated with high vascular density are correlated with an increased risk of developing breast cancer. Traditionally, the mechanism(s) controlling the development of tumor vasculature and perfusion were thought to be endothelial cell-lined vascular networks [3]. Thus, efforts to reduce the growth and spread of breast cancer focused on the mechanism(s) of angiogenesis by which tumors establish a blood supply for survival, growth, and metastasis [4].

However, recent studies have found that some highly aggressive tumor cells generate vessel-like channels in the absence of endothelial cells or fibroblasts [5, 6]. This phenomenon is termed vasculogenic mimicry (VM). These channels are thought to provide a new mechanism of perfusion and a dissemination route within the tumor that functions either independently of, or simultaneously with, angiogenesis [7-10]. More importantly, previous studies have demonstrated that aggressive breast cancer is characterized by VM channels and Periodic Acid-Schiff (PAS)-positive patterns, and is associated with poor clinical prognosis [11]. Conventional anti-vascular therapies targeting endothelial cells have no significant effects on tumors with VM, and the overall survival rates for most cancer patients are not significantly prolonged [12, 13]. Therefore, dual targeting of endothelial sprouting angiogenesis and tumor cell-mediated VM may be required to overcome current clinical problems with anti-angiogenic therapy and improve clinical outcome.

Claudins are crucial structural and functional components of tight junctions, which are essential for holding endothelial and/or epithelial cells together, regulating paracellular permeability and maintaining cell polarity [14, 15]. In humans, 24 members of the claudin family have been identified [14, 16]. These proteins contain four transmembrane domains, which create two extracellular loops that direct homotypic claudin interactions. Numerous claudin members are expressed by different breast cancer subtypes. In aggressive breast cancer, expression of claudin-3 and -4 have been shown to be upregulated, while claudin-1 and -7 proteins were downregulated, suggesting that individual claudin family members play different roles in breast carcinogenesis [17-19]. For example, claudin-4-expressing ovarian epithelial cells exhibit upregulation of several genes encoding pro-angiogenic cytokines, and can induce angiogenesis both *in vitro* and *in vivo* in mice [20]. Claudin-2 has been shown to mediate tumor cell/hepatocyte interactions and the ability of breast cancer cells to form liver metastases *in vivo* [21]. Aggressive breast cancer cells may also express many specific endothelial cell (EC) markers, including thrombin receptor, TIE-2, VE-cadherin, VEGF, CD31, and CD34 [22-27]. Taken together, these studies reveal the diverse roles of claudins in tumor cell-mediated neovascularization.

Although the vessel-like channels originating from aggressive tumor cells are substantially different from endothelial vessels, it is possible that highly aggressive breast cancers are predisposed to form VM more easily than non-aggressive forms because of their endothelial-like characteristics [28]. We therefore hypothesized that overexpression of certain claudin members may contribute to VM formation. In the present study, we analysed the possible relationship of claudin-2, -3, -4, -6, -7, and -17 expression and VM formation in two breast cancer cell lines, aggressive MDA-MB-231 and non-aggressive MCF-7 cells, and the human umbilical vein endothelial cell line (HUVEC). We then assessed whether overexpression of claudin or inhibition of claudin function by treatment of these cells with monoclonal antibodies (mAbs) or targeted silencing using short hairpin RNA (shRNA), promoted or inhibited vascular channel formation, respectively. The aims of this study were to compare the ability of human breast cancer cells expressing high levels of claudins to form vascular channels on three-dimensional matrigel cultures, and to further identify candidate proteins involved in VM formation.

## RESULTS

### Aggressive breast cancer cells exhibit a stronger ability to form VM than non-aggressive cells *in vitro*

**Figure 1:**
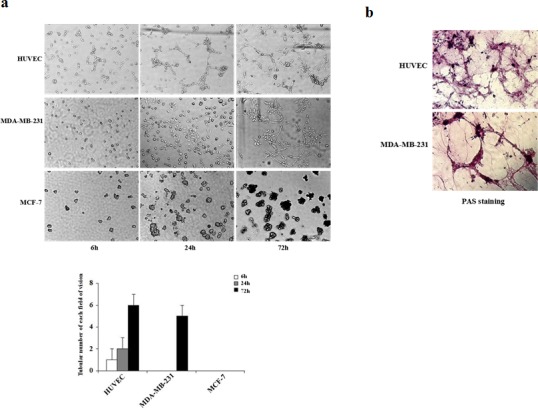
Kinetics of vascular channel formation in HUVEC, MDA-MB-231, and MCF-7 cells (a) HUVEC, MDA-MB-231, and MCF-7 cells were plated on matrigel, and images were acquired using phase-contrast microscopy at the times indicated. HUVEC cells started to form vascular channels 6 h after plating whereas MDA-MB-231 cells formed vascular channels at 24 h. Well-defined patterned networks were observed by 72 h. MCF-7 cells failed to form patterned networks. (b) Periodic Acid-Schiff (PAS) staining of HUVEC and MDA-MB-231 cells plated on matrigel for 72 h to identify secreted extracellular matrix. Pink staining identifies glycogen and related mucopolysaccharides secreted by cells to form the extracellular matrix-rich vascular channels.

Three-dimensional cultures were performed to evaluate the differential abilities of aggressive MDA-MB-231 and non-aggressive MCF-7 breast cancer cell lines to form vascular channels (Fig. 1). HUVEC cells were used as a positive control. Cells were observed by phase-control microscopy during the 72-h incubation period. As shown in Fig. 1, we observed significant differences in the efficiency of vascular network formation between the three cell lines when plated on matrigel. The highly aggressive MDA-MB-231 cells generated similar patterns compared with HUVEC cells, consisting of a tubular network (Fig. 1a). These tubular structures started to form at 24 h, later than those of HUVEC cells (at 6 h), and were clearly visible by phase microscopy after 72 h (Fig. 1a). In contrast, no tubular networks were observed following incubation of non-aggressive MCF-7 cells for 72 h on matrigel (Fig. 1a), and channels were undetectable up to 120 h after plating (data not shown). To further investigate the matrix-associated vascular channels formed by HUVEC and MDA-MB-231 cells, we next performed PAS staining, which identifies glycogen and related mucopolysaccharides secreted by these cells to form the extracellular-matrix-rich channels. At 72 h, both HUVEC and MDA-MB-231 cells exhibited strong PAS positivity (Fig. 1b). These results provide direct evidence that aggressive breast cancer cells are more potent in VM formation than non-aggressive cells.

### Claudin-4 and -6 expression is aberrantly upregulated in aggressive breast cancer cells

**Figure 2:**
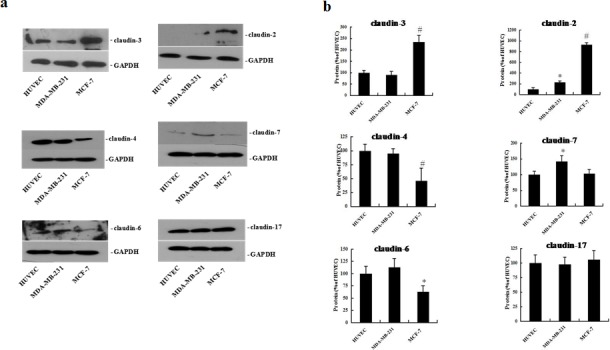
Expression of claudin-2, -3, -4, -6, -7, and -17 proteins in HUVEC, MDA-MB-231, and MCF-7 cells HUVEC, MDA-MB-231, and MCF-7 cells were plated on matrigel for 72 h. Western blot analysis of claudin proteins was performed using whole cell protein lysate. (a) Representative blots of claudin-2, -3, -4, -6, -7, and -17 (b) The corresponding expression levels are shown as bar graphs. Claudin protein levels in HUVEC cells were defined as 1. Data represent the mean + SD (n=3), *: p < 0.05 compared with HUVEC cells. #: p < 0.01 compared with HUVEC cells.

Because claudins participate in homo- and heteromeric interactions between adjacent cells, we next investigated the expression levels of six claudin family members (claudin-2, -3, -4, -6, -7, and -17) in the three cell lines by western blotting analysis. As shown in Fig. 2, we observed expression of all six claudin proteins in both the highly aggressive MDA-MB-231 cells and the non-aggressive MCF-7 cells. In contrast, only five claudin members (claudin-3, -4, -6, -7 and -17) were expressed in HUVEC cells (Fig. 2a). Notably, levels of claudin-3, -4, -6 and -17 were comparable between aggressive MDA-MB-231 cells and HUVEC cells, while non-aggressive MCF-7 cells expressed significantly lower levels of claudin-4 and -6 (Fig. 2b). Conversely, high levels of claudin-2 and -3 proteins were observed in MCF-7 cells (Fig. 2b). These results indicate that overexpression of claudin-4 and -6 are associated with aggressive potential in a breast cancer cell line, suggesting that these proteins may be implicated in VM formation in this *in vitro* cell model.

### Inhibition of claudin-4 but not claudin-6 using mAbs significantly inhibits VM formation *in vitro*

**Figure 3:**
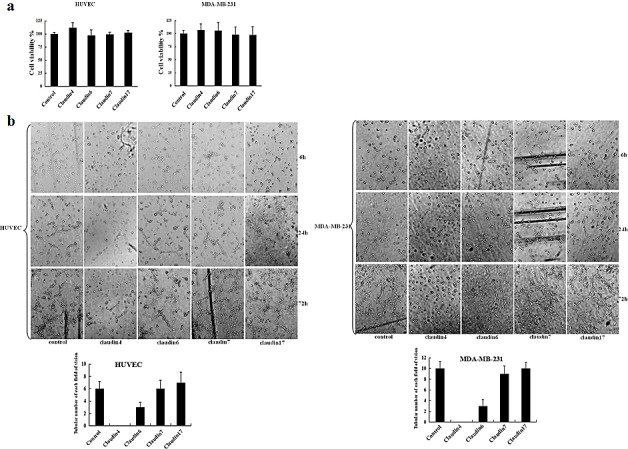
Effects of claudin blocking antibodies on cell proliferation, morphology, and tubule formation (a) MTT assay was used to assess the effects of different blocking antibodies on the proliferation of HUVEC and MDA-MB-231 cells. HUVEC and MDA-MB-231 cells were plated in 96-well plates. Medium containing claudin-2, -3, -4, -6, -7, or -17 blocking antibodies (1 μg/mL) were added to the 96-well plates. An equivalent volume of mouse IgG1 control antibody was used as a control. Data represent the mean + SD (n=3). *: p < 0.05 compared with controls. (b) HUVEC and MDA-MB-231 cells were also cultured in matrigel. Images were acquired by phase-contrast microscopy at the times indicated.

To determine whether aberrant expression of claudins contribute to VM formation, HUVEC and MDA-MB-231 cells were treated with mAbs targeting claudin-4, -6, -7, or -17 for up to 72 h. Toxicity was then evaluated by examining cell morphology and MTT assays. No change in cell proliferation was observed following treatment of the two cell lines with different blocking antibodies after 72 h (Fig. 3a). In two-dimensional cultures, treatment with claudin-4 mAb or antibodies targeting other claudin members did not significantly alter the morphology of HUVEC and MDA-MB-231 cell lines (data not shown). In contrast, treatment of cells with different blocking antibodies had differential effects on tubular formation in three-dimensional cultures (Fig. 3b). Treatment of cells with a claudin-4 blocking antibody significantly inhibited tubular formation in MDA-MB-231 cells at 72 h compared with control, while a claudin-6 blocking antibody had minimal effects on the cell lines (Fig. 3b). No effects on tubular formation were observed following treatment with claudin-7 or claudin-17 blocking antibodies (Fig. 3b). Similar effects were observed in HUVEC cells following treatment with individual blocking antibodies (Fig. 3b). These findings demonstrate a significant correlation between claudin-4 and VM formation in breast cancer cells.

### Inhibition of VM formation following specific targeting of claudin-4 in MDA-MB-231 cells by shRNA

**Figure 4:**
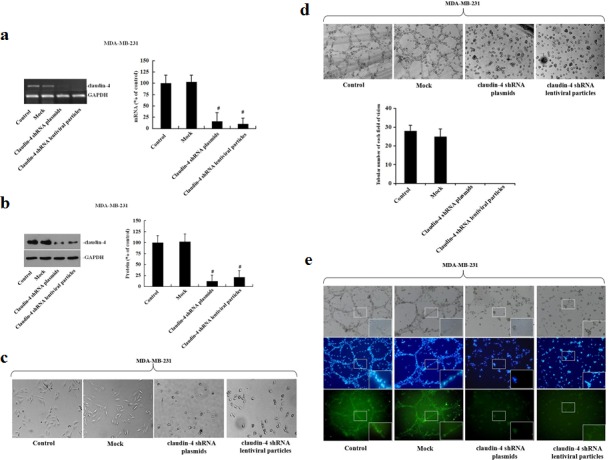
Analysis of vascular channel formation following stable transfection of MDA-MB-231 cells with claudin-4-specific shRNA plasmids or lentiviral particles MDA-MB-231 cells grown to 60% confluence were transfected with claudin-4 specific plasmids, lentiviral particles, or control shRNAs. Transduced cells were selected with puromycin (2 μg/ml) after 24 h. (a) Levels of *claudin-4* mRNA were examined by nested reverse-transcription-polymerase chain reaction (RT-PCR). *Claudin-4* mRNA levels were determined by densitometry and normalized to *GAPDH*. Changes are expressed as a percentage of control. #: p < 0.01 compared with controls. (b) Expression of claudin-4 protein was assessed by western blot and quantification is represented in bar graphs. Defined claudin-4 protein of the control group as 1, other groups compared with it. Data represent the mean + SD (n=3). #: p < 0.01 compared with controls. (c) Morphological characteristics of MDA-MB-231 cells were visualized by phase-contrast microscopy. (d) Changes in vascular channel formation in MDA-MB-231 cells. MDA-MB-231 cells were plated on matrigel, and images were acquired by phase-contrast microscopy at the times indicated. (e) Representative confocal images of non-transfected, transfected blank vector and two stably transfected MDA-MB-231 cells. Following fixation, the cells were stained with a claudin-4 antibody (green) and DAPI (blue).

To verify our *in vitro* results obtained using the claudin-4 mAb, we silenced the expression of claudin-4 protein using shRNA technology. MDA-MB-231 cells were transfected with claudin-4-specific shRNA plasmids or transduced with lentiviral particles, and stable clones were isolated with puromycin. VM formation potential was subsequently determined in matrigel assays. As shown in Fig. 4, transfection of MDA-MB-231 cells with shRNA plasmids or lentiviral particles induced a marked decrease in *claudin-4* gene expression as assessed by nested RT-PCR (Fig. 4a) and also at the protein level (Fig. 4b). In two-dimensional cultures, claudin-4 knockdown in MDA-MB-231 cells led to substantial morphological changes, with a transition from a long shuttle to cobblestone-like shape (Fig. 4c). While mock-transfected cells clustered together in groups, claudin-silenced cells appeared more isolated (Fig. 4c). Notably, silencing of claudin-4 significantly reduced the number of tubular channels formed by MDA-MB-231 cells compared with sh-control cells in three-dimensional cultures (Fig. 4d). Immunofluorescence analysis identified claudin-4 staining at the cell membrane and in the cytoplasm of MDA-MB-231 cells (Fig. 4e). In contrast, expression of claudin-4 was significantly inhibited at the membranes of cells transfected with claudin-4 specific shRNA plasmids or transduced with lentiviral particles (Fig. 4e). Taken together, these results demonstrate that knockdown of claudin-4 has a significant functional effect on VM formation in MDA-MB-231 cells.

### Overexpression of claudin-4 enhances VM formation in MCF-7 cells

**Figure 5:**
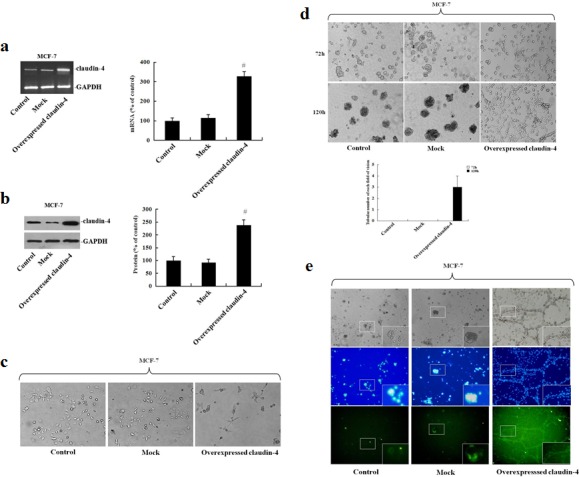
Analysis of vascular channel formation following stable transfection of MCF-7 cells with pEGFP-N1-CLDN4 MCF-7 cells were stably transfected with pEGFP-N1-CLDN4. Cells were selected in the presence of G418 (200 μg/mL) and clonal cell lines were established. (a) Clones were maintained in medium containing G418 and screened for claudin-4 overexpression by RT-PCR. *Claudin-4* mRNA levels were assessed by densitometry and normalized to *GAPDH*. Changes are expressed as a percentage of the control. #: p < 0.01 compared with controls. (b) Claudin-4 protein levels were assessed by western blot and quantification is represented in bar graphs. Defined claudin-4 protein of the control group as 1, other groups compared with it. Data represent the mean + SD (n=3). #: p < 0.01 compared with controls. (c) Morphological characteristics of MCF-7 cells were visualized by phase-contrast microscopy. (d) Changes in vascular channel formation in MCF-7 cells. MCF-7 cells were plated on matrigel, and images were acquired by phase-contrast microscopy at the times indicated. (e) Representative confocal images of non-transfected, transfected blank vector and stably transfected MCF-7 cells. Following fixation, cells were stained with a claudin-4 antibody (green) and DAPI (blue).

To further confirm the role of claudin-4 in VM formation, we next constructed a cell line overexpressing claudin-4 by transfecting MCF-7 cells with a claudin-4 expression vector. Overexpression of *claudin-4* mRNA and protein was confirmed by nested RT-PCR (Fig. 5a) and western blot, respectively (Fig. 5b). Claudin-4 overexpression led to a significant change in the morphology of MCF-7 cells in two-dimensional cultures (Fig. 5c), with cells acquiring a scattered phenotype. Overexpression of claudin-4 also led to an increase in the formation of VM compared with empty vector control cells (Fig. 5d). Immunofluorescence analysis of MCF-7 cells stably transfected with claudin-4, revealed expression predominantly at the cell membrane compared with control cells (Fig. 5e). Taken together, these results indicate that claudin-4 may be critical for VM formation in breast cancer cell lines.

## DISCUSSION

Recent reports support the existence of a non-angiogenic as well as an angiogenic pathway in breast cancer metastasis, which may complicate treatment strategies [28, 29]. In the present study, we aimed to explore the potential relationship between claudins and VM in breast cancer. To the best of our knowledge, this is the first study to report that expression of a relatively understudied, cell junctional molecule, claudin-4, promotes VM in breast cancer. Mechanistically, this occurs by enhancing the interactions between tumor cells, suggesting that dual targeting of endothelial sprouting angiogenesis and tumor cell-mediated neovascularization may improve current anti-cancer therapies.

VM channels are considered to function either independently of or simultaneously with angiogenesis [7-10]. HUVEC cells exhibit angiogenic capability and are capable of forming tubular structures [30, 31]. Hence, in our study, HUVEC cells were used as a positive control cell model for *in vitro* VM. Two breast cancer cell lines, highly aggressive MDA-MB-231 cells and non-aggressive MCF-7 cells, were selected to investigate the cellular mechanisms of tumor vasculogenesis. We observed that highly aggressive MDA-MB-231 cells formed patterned, matrix-associated vascular channels *in vitro* (Fig. 1). In contrast, non-aggressive MCF-7 cells were not capable of generating patterned vascular channels (Fig. 1). Previous studies have shown that aggressive human breast cancer cell lines, MDA-MB-231 and MDA-MB-435, are capable of developing vascular channels when plated on three-dimensional matrigel cultures, in contrast to non-aggressive MCF-7 and ZR-75-1 cell lines [32]. Thus, our findings are in agreement with previous reports demonstrating the unique ability of highly aggressive breast cancer cells, but not poorly aggressive cells, to form vascular-like structures.

Aggressive tumor cells exhibit a high degree of plasticity, indicative of a multi-potent phenotype, similar in many respects to embryonic stem cells [33-35]. Molecular profiling of cells exhibiting the tumor cell VM phenotype, identified a number of highly upregulated genes associated with embryonic progenitors and endothelial cells [35]. Highly aggressive tumor cells capable of forming VM channels also express genes implicated in angiogenesis. Despite high expression of VEGF, VEGFR1, VEGFR2, bFGF, bFGFR, COX-2, von Willebrand factor, VE-cadherin, and laminin-5γ2 in these cells, VM does not depend on tumor angiogenesis [36]. Moreover, these cells express matrix metalloproteinases MMP-1, -2, -9, and -14, which are capable of modifying the extracellular matrix, a necessary step for classical angiogenesis [37]. Not surprisingly, a number of markers expressed by MDA-MB-231 cells, including the thrombin receptor, TIE-2, CD31, VEGF, and bc-48, have been associated with endothelial cells [38-42]. These studies suggest that vascular channel formation by aggressive breast cancer cells may follow the same regularity as formation of blood vessels by endothelial cells. Thus, highly aggressive breast cancer cells may imitate the behavior of endothelial cells and initiate formation of vascular channels.

Claudins are a family of transmembrane proteins that directly mediate endothelial and/or epithelial cell-cell adhesion [43, 44]. Studies have identified distinct claudin expression patterns within specific subtypes of breast cancer. Previous studies have shown that aggressive breast cancer is characterized by upregulation of claudin-3 and -4 and downregulation of claudin-1 and -7 proteins [17-19]. In our study, we identified abnormal overexpression of two claudin members, claudin-4 and -6, in aggressive breast cancer cells. Furthermore, the roles of these two claudins were defined using blocking antibodies to observe effects on VM formation. We demonstrate that claudin-4 or claudin-6 blocking antibodies have different effects on VM formation in aggressive breast cancer cells *in vitro*. Specific inhibition of claudin-4 using mAbs led to complete inhibition of vascular channel formation in aggressive breast cancer cells, while this effect was minimal using claudin-6 blocking antibody (Fig. 3). Treatment of HUVEC cells with claudin-4 or -6 mAbs yielded similar results (Fig. 3). Previous studies have shown that claudin-4 is associated with prognosis in breast or bladder cancer, and plays a role in breast cancer cell invasiveness and metastatic potential in addition to inducing angiogenic factors in ovarian cancer [20, 45-47]. Thus, further studies investigating the overexpression of claudin-4 in aggressive breast cancer cells may help to elucidate the molecular mechanisms underlying the channel-forming ability of these highly aggressive tumor cells.

The results prompted us to conduct a preliminary functional analysis to investigate the effect of specifically inhibiting or overexpressing claudin-4 in aggressive and non-aggressive breast cancer cells, respectively, on VM. Transfection of aggressive MDA-MB-231 cells with shRNA specific to claudin-4 or transduced the cells with lentiviral particles inhibited vascular formation. Conversely, transfection of non-aggressive MCF-7 cells with a claudin-4 over-expression vector, induced formation of patterned vascular channels. These results support our hypothesis that claudin-4 serves as a required cellular factor necessary for VM formation. Furthermore, immunocytochemistry showed that membranous claudin-4 protein was significantly associated with vascular channel formation. Mitchell *et al.,* demonstrated that increased expression of claudin-4 was sufficient to increase alveolar epithelial cell barrier function [48]. These results suggest that claudin-4 may function to increase and maintain tumor cell-cell contact, eventually contributing to VM formation.

In conclusion, our data demonstrate that aggressive breast cancer cells are more potent inducers of VM formation than non-aggressive cells. Moreover, we show that claudin-4 is associated with the formation of VM in human breast cancer cells. Overexpression of claudin-4 promoted an increase in VM formation *in vitro*. Conversely, decreased claudin-4 inhibited VM formation. These observations enhance our understanding of the mechanism of VM formation in aggressive breast cancer, and may assist the development of better therapeutic strategies for the treatment of this disease.

## MATERIALS AND METHODS

### Cell culture

The human breast cancer cell lines, MDA-MB-231 and MCF-7 and human umbilical vascular endothelial cell line, HUVEC were purchased from the American Type Culture Collection (ATCC, Manassas, VA, USA). Briefly, cells were cultured in Dulbecco's modified Eagle medium (DMEM) supplemented with 10% fetal calf serum (FCS), penicillin (100 U/mL), and streptomycin (100 μg/mL), and incubated in a humidified atmosphere with 5% CO_2_ in air at 37 °C.

### Three-dimensional cultures and tubule formation

Six-well plates were pre-coated with Matrigel (BD Biosciences, Franklin Lakes, NJ, USA) and incubated at 37 °C for 30 min. Cells (4×10^4^ cells/well) were seeded in 6-well plates and maintained in DMEM medium supplemented with FCS during the incubation. For blocking antigen, medium containing mouse monoclonal anti-claudin-2, -3, -4, -6, -7 or -17 antibodies (1 μg/mL, Genetex, Irvine, CA, USA) were added to other 6-well plates. An equivalent volume of mouse IgG1 control antibody (Abcam, Cambridge, UK) was used as a negative control. Following incubation for 6, 24 and 72 h, tubule formation was observed and photographed using a phase-contrast microscope (Leica Microsystems, Wetzlar, Germany).

### Proliferation assay

Cells (4×10^4^ cells/well) were seeded in 96-well plates in quintuplicate and incubated with individual mouse monoclonal antibodies (1 μg/mL) or mouse IgG1 negative control antibody at 37 °C. After 3 days, proliferation was measured by MTT assay (Invitrogen, Carlsbad, CA, USA) according to the manufacturer's guidelines.

### Periodic acid schiff (PAS) staining

Tumor three-dimensional cultures were stained with PAS according to standard procedures. Briefly, cells were fixed for 1 h at 4 °C in 4% formaldehyde. Cells were then pre-treated for 5 min with 1% periodic acid solution (Sigma-Aldrich, St. Louis, MO, USA) followed by washing for 1 min in tap water. Cells were then incubated with Schiff's reagent (Sigma-Aldrich) for 15 min at room temperature and plates were subsequently washed for 10 min in tap water with gently shaking. Stained cells were photographed under a phase-contrast microscope (Leica Microsystems).

### Immunofluorescence staining

Cells were plated on chamber slides and fixed in ice-cold 100% methanol for 20 min. Cells were stained with claudin-4 primary antibody (1:100, Genetex, Irvine, CA, USA) and secondary fluorescein isothiocyanate-conjugated antibody (Santa Cruz Biotechnology, Santa Cruz, CA, USA). After immunolabeling, cells were washed, stained with DAPI (Sigma-Aldrich), and viewed by fluorescence microscopy (Leica Microsystems).

### Plasmids, transient transfection, and generation of stable cell lines

The human claudin-4 cDNA was amplified using the sense primer, CLDN4-1: 5′-CAAGATCTCCGTGGACGCTGAACAATGG-3′ [5′ Bgl II site underlined] and the antisense primer, CLDN4-2: 5′-CAGGTACCAGTGGAGCCGTGGCACCTTA-3′ [Kpn I site underlined] and human cDNA as a template. The reaction product was purified by agarose gel electrophoresis and cloned into the pGEM-T easy vector (Promega, Madison, WI, USA). Nucleotide sequences of the PCR products were verified by dideoxynucleotide chain-termination sequencing using a Li-COR 4000 L automated DNA sequencer. The resulting plasmid, pCLDN4, was digested with Bgl II and Kpn I, and the fragment containing the full-length *CLDN4* cDNA was ligated into pEGFP-N1 (Invitrogen, Carlsbad, CA, USA) to generate pEGFP-N1-CLDN4. Transfection was performed using Lipofectamine (Invitrogen) according to the manufacturer's protocol. 24 h after transfection, cells were selected in the presence of G418 (200 μg/mL, Invitrogen), and clonal cell lines were established. Clones were maintained in DMEM medium containing G418 and screened for claudin-4 overexpression by nested reverse-transcription-polymerase chain reaction (RT-PCR) and western blot analysis.

### shRNA knockdown

MDA-MB-231 cells were plated in 6-well plates (4×10^4^ cells/well) and grown to 60% confluence. Cells were transfected with claudin-4 specific shRNA plasmid, lentiviral particles or control shRNA (Santa, Carlsbad, CA, USA) using shRNA plasmid transfection reagent, in accordance with the manufacturer's instructions. Briefly, for each well, 10 μL transfection reagent was incubated with 90 μL transfection medium for 5 min. shRNA plasmids were diluted in transfection medium, added to the transfection media (final shRNA concentration 100 nmol/L), and subsequently added to the wells. For lentiviral particles, first removed media from plate wells and replaced with 1ml of polybrene (Santa, Carlsbad, CA, USA)/media mixture per well, then infected cells by adding the shRNA lentiviral particles. Following 24 h incubation, cells were treated with puromycin (2μg/ml) to select stably transduced cells and cell colonies were selected and expanded for downstream experiments.

### Semi-quantitative nested RT-PCR

Total RNA was isolated from cell lines using Trizol-GenClean (TianGene, Beijing, China) according to the manufacturer's protocol. RT-PCR was performed using the RevertAid™ First Strand cDNA Synthesis Kit (TianGene). cDNA was synthesized from total RNA using oligo(dT)18 primers and RevertAid M-MuLV Reverse Transcriptase. PCR reactions were performed in a 25-μL volume comprising PCR buffer (1×), cDNA (50 ng), forward and reverse primers (25 pmol each), MgCl_2_ (1.5 mM), dNTPs (0.2 mM each), and Taq DNA polymerase (3.0 U). Amplification was performed by denaturation at 95 °C for 5 min, followed by 25 cycles of 95 °C for 60 s, 66 °C for 60 s, and 72 °C for 60 s, and a final extension at 72 °C for 10 min. Amplification for second-round PCR and *GAPDH* were performed in the same tube. The RT-PCR products were analyzed by 1.2% agarose gel electrophoresis and stained with ethidium bromide.

### Western blot analysis

Cells were homogenized in RIPA buffer supplemented with protease inhibitors (Roche, Basel, Switzerland). Total protein (10 μg) was separated by 12% sodium dodecyl sulfate-polyacrylamide gel electrophoresis (SDS-PAGE) and transferred by electroblotting onto nitrocellulose membranes. The blots were blocked by incubation with 5% blocking reagent in a solution of Tris-buffered salt with Tween-20 (TBS-T) for 1 h at room temperature, then incubated with primary antibody (1:1000) overnight at 4 °C. After washing with TBS-T, blots were incubated in secondary antibody conjugated horseradish peroxide (1:1000) for 1 h at 37 °C. Following extensive washing, the complexes were visualized using the West Pico chemiluminescent kit (Pierce, Rockford, IL, USA).

### Statistical analysis

Data are expressed as the mean ± standard deviation (SD). Differences between the means were determined by one-way analysis of variance followed by a least-significant-difference test for multiple comparisons. A probability value of p < 0.05 was regarded to be statistically significant.
